# JMJD3 in the regulation of human diseases

**DOI:** 10.1007/s13238-019-0653-9

**Published:** 2019-11-07

**Authors:** Xiangxian Zhang, Li Liu, Xia Yuan, Yuquan Wei, Xiawei Wei

**Affiliations:** Laboratory of Aging Research and Nanotoxicology, State Key Laboratory of Biotherapy, National Clinical Research Center for Geriatrics, West China Hospital, Sichuan University, Chengdu, 610041 China

**Keywords:** histone methylation, JMJD3, epigenetics, human diseases

## Abstract

In recent years, many studies have shown that histone methylation plays an important role in maintaining the active and silent state of gene expression in human diseases. The Jumonji domain-containing protein D3 (JMJD3), specifically demethylate di- and trimethyl-lysine 27 on histone H3 (H3K27me2/3), has been widely studied in immune diseases, infectious diseases, cancer, developmental diseases, and aging related diseases. We will focus on the recent advances of JMJD3 function in human diseases, and looks ahead to the future of JMJD3 gene research in this review.

## **INTRODUCTION**

**Figure 1:**
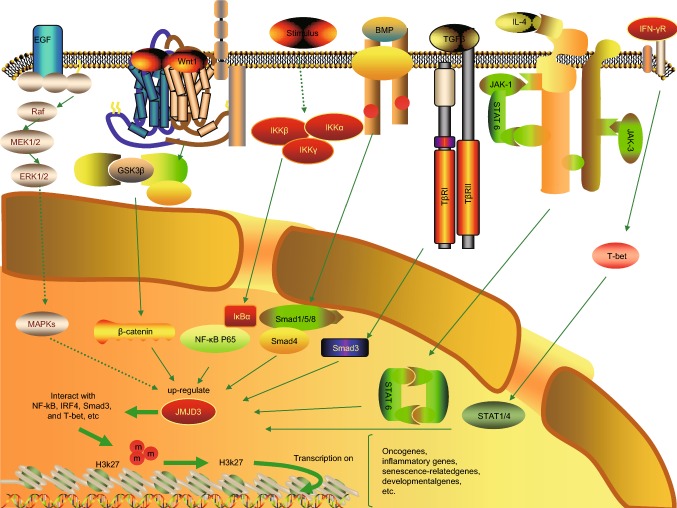
**A schematic figure describing the regulatory network associated with JMJD3**. JMJD3 can be upregulated via distinct signalling pathways, i.e., MAPK signaling, Wnt signalling, NF-κB signaling, BMP signalling, TGF-β signalling, IL-4-STAT6 signaling, and T-bet signaling. Then JMJD3 is recruited to the chromatin via interacting with several transcription factors. JMJD3 activates the transcription of oncogenes, inflammatory genes, developmental genes by demethylating the repressive H3K27me3 markers on their promoters and gene bodies. The induction of JMJD3 modifies the responses during the occurrence and development of various kinds of human diseases

A substantial literature indicated that methylation, one of the most important post-translational modifications of histone, was quite important in the regulation of gene expression (Mozzetta et al. 2015). Histone methylation had been reported to participate in the epigenetic control of health and diseases (Feinberg 2018). H3K27me3 (trimethylated lysine 27 on histone 3) is a critical epigenetic event frequently associated with gene repression (Wiles and Selker 2017). When H3K27 is trimethylated, it typically means that gene expression is inhibited; and when H3K27 is monomethylated, it typically means that gene expression is activated (Barski et al. 2007). JMJD3, with another name lysine-specific demethylase 6B (KDM6B), is a member of JmjC histone demethylases family that specifically demethylate and trimethylate lysine at position 27 of H3 protein to regulate correlation gene expression (Swigut and Wysocka 2007). JMJD3 also mediates the interactions of chromatin modifiers to activate targeted genes independent of its demethylase activity (Salminen et al. 2014). JMJD3 contains a JmjC catalytic domain and a C-terminal segment, and consists 1,679 amino acids (Xiang et al. 2007). In normal conditions, the expression of JMJD3 is in low level in the organization, but a variety of cellular stress stimulation can strongly induce the expression of JMJD3 (Fig. 1) (de Santa et al. 2007; Lee et al. 2014a, b; Arcipowski et al. 2016). JMJD3 plays a critical role in development, physiology and diseases (Salminen et al. 2014; Burchfield et al. 2015; Arcipowski et al. 2016). GSK-J4 is a JMJD3 specific small-molecule inhibitor, and it has been used in selective pharmacological intervention of JMJD3 in several mouse disease models (Kruidenier et al. 2012). We will review the role of JMJD3 in the occurrence and development of various kinds of human diseases such as immune diseases, infectious diseases, cancer, developmental diseases, aging related diseases, and the mechanisms.

## **STRUCTURE OF JMJD3**

**Figure 2:**
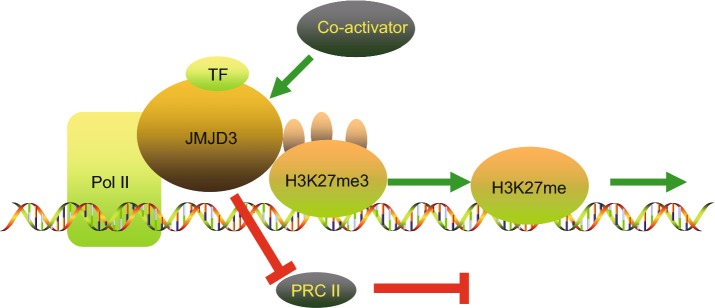
A graphical illustration on the mechanisms of JMJD3 in the regulation of enhancing gene transcription. JMJD3 promotes gene transcription in a demethylase-dependent or independent manner. JMJD3 enhances the initiation of transcription through three ways. Firstly, JMJD3 demethylate H3K27me3 on the promoter of targeted genes. Secondly, JMJD3 releases suppressive PRC2 complexes. Thirdly, JMJD3 binds to the Pol II elongation complex, enhancing its progression through H3K27me3-enriched gene bodies to enhance transcription elongation. This is the demethylase-dependent process. JMJD3 recruits co-activators to targeted gene promoters by interacting with both the co-activator and transcription factors in gene promoters. This co-activation process does not require demethylase activity. This is the demethylase-independent manner

JMJD3 is a member of the UTX/UTY JmjC-domain protein subfamily. UTX and UTY genes of the mammal separately reside on the X and Y chromosome (Klose et al. 2006). Structurally, JMJD3 sequence is highly homology with UTX and UTY, but JMJD3 has no TPR domains compared with UTX and UTY. UTX and JMJD3 have H3K27-specific demethylase activity, except for UTY. UTX has the ability to demethylate H3K27me1/2/3, and JMJD3 has the ability to demethylate H3K27me2/3 (Hong et al. 2007). Differing from the fact that the function of the UTX/UTY proteins is largely unknown, JMJD3 has been proved to be widely involved in the key gene expression during physiological development and diseases occurrence and development. Human JMJD3 gene is located at 17p13.1 and encodes a polypeptide containing 1,682 amino acid residues (Hu et al. 2017). A C-terminal segment and JmjC domain constitute the main structure of JMJD3. The C-terminal segment of JMJD3 is embedded with a Zn^2+^ -coordinated GATA-like (GATAL) domain of novel topology, and the GATAL domain is flanked by a four-helix bundle formed by α-helical segments (Kruidenier et al. 2012). The JmjC domain is catalytically active. Eight β-sheets of the JmjC domain coordinates Fe^2+^ and α-ketoglutarate cofactor play as an enzymatically active centre. In the enzymatically active centre, two amino-acid residues bind to the α-ketoglutarate cofactor, and three amino-acid residues bind to the Fe^2+^ cofactor. Highly reactive oxoferryl species are considered to be produced in the JmjC domain, and catalyse histone demethylation by hydroxylating the methylated substrate (Chen et al. 2006). The molecular mechanisms of JMJD3 in the regulation of gene transcription are shown in Fig. 2. Specially, JMJD3 also acts as a transcription factor. JMJD3 activates the transcription of target genes by interacting with co-activators, which is independent of its demethylase activity. JMJD3 recruits co-activators, and interacts with co-activators and transcription factors in the promoter region of the target gene to activate transcription (Salminen et al. 2014). At present, non-demethylase action of JMJD3 still needs further study.

## **JMJD3 IN IMMUNE DISEASES**

JMJD3 is a double-edged sword in immune diseases including autoimmune diseases for it contributes to both pro-inflammatory and anti-inflammatory responses by activating different transcription factors in gene promoters. JMJD3 is up-regulated by many inflammatory mediators and stress inducers through different signaling pathways. Then, JMJD3 interacts with certain transcription factors, e.g., nuclear factor-kappa B (NF-κB), T-box, interferon regulatory factor 4 (IRF4), SMAD3, and is recruited to chromatin. JMJD3 controls those crucial inflammation associated transcription factors through either demethylating H3K27me3 or in manners unrelated to demethylase, and play a role in inflammatory diseases. JMJD3 has been proved to play a pro-inflammation role in the epigenetic regulation of innate immunity during early sepsis (Chen et al. 2018), wound healing (Odorisio 2016; Na et al. 2016), neuroinflammation and neuropathic pain (Penas and Navarro 2018), periodontitis (Puyu W), Kawasaki disease (Mei et al. 2017), blood-spinal cord barrier disruption (Lee et al. 2012), rheumatoid arthritis (RA) (Jia et al. 2018), osteoarthritis (Yapp et al. 2016), encephalomyelitis (EAE) (Doñas et al. 2016), acute myocardial infarction (MI) (Yan et al. 2013), exercise-induced bronchoconstriction (EIB) (He et al. 2013), and systemic lupus erythematosus (Zhang et al. 2011), and its specific inhibitor GSK-J4 could inhibit the development of these inflammatory diseases through inhibiting the production of inflammatory cytokines in immune cells via various signaling pathways. It has been reported that inhibition of JMJD3 through gene silencing or JMJD3 specific inhibitor GSK-J4 significantly relieved arthritis in collagen-induced arthritis (CIA) mice; clinical manifestation of EAE was relieved by the adoptive transfer of DCs pretreated with GSK-J4 in EAE mice and the level of inflammatory CD4^+^ T cells in the central nervous system was decreased; in systemic lupus erythematosus (SLE), B cell overstimulation and T cell overactivation were considered to be associated with the high level of H3K27me3 at the HPK1 promoter, and the low level of JMJD3 up-regulation in post-MI heart promoted the M2 transformation of macrophages, and controlled the inflammation process in post-MI heart. JMJD3 performs opposite functions under different environmental stimuli. JMJD3 is necessary for M2 polarization of the macrophage. M2 macrophage mainly plays anti-inflammation roles. JMJD3 was found regulated by adiponectin (APN) to contribute to M2 macrophage polarization in obesity, thereby attenuating chronic inflammation. A schematic figure representing the regulatory network of JMJD3 in inflammation diseases is shown in Fig. 1.

### **Pro-inflammatory effect of JMJD3**

A large amount of studies have showed that JMJD3 enhances pro-inflammatory genes expression, which is typically associated with the activation of NF-κB signaling, STAT signaling, TGF-β/SMAD3 signaling, and T-bet signaling (de Santa et al. 2009; Das et al. 2012; Hanisch 2014).

#### *NF-κB signaling*

The activation of NF-κB as an early and central event in inflammation (Pasparakis 2009). The transcription factors of the NF-κB family directly control the induction of a very large fraction of the inflammatory transcriptome (Zhang et al. 2017). Molecular studies have revealed the reason why JMJD3 transcription could be rapidly induced by NF-κB. It is because that the promoter sequences from the first coding exon of JMJD3 contain two conserved κB sites (de Santa et al. 2007). JMJD3 is also reported as a stimulator of NF-κB, and forms a positive feedback loop on promoting each other’s transcription (Wei et al. 2013). These findings confirm that JMJD3 plays important roles in NF-κB dominated inflammatory stimulation. NF-κB-JMJD3 pathway is crucial in Lipopolysaccharides (LPS)-mediated inflammation. LPS treatment triggered JMJD3 to bind to the transcription promoter of thousands of crucial inflammatory genes in bone marrow-derived macrophages. However, the transcription of numerous inflammatory genes were inhibited after the knockdown of JMJD3 expression (de Santa et al. 2009; Das et al. 2012). NF-κB-JMJD3 signaling enhanced the expression of IL-1β, TNF-α, IL-6, ICAM-1 and MMP-9 in LPS treated human umbilical vein endothelial cells (HUVECs) and might contribute to vascular inflammation and atherosclerosis (Chen et al. 2017; Yu et al. 2017). JMJD3 was found to activate the Nox4 autophagy signalling to promote the neointimal formation after vascular injury, and was considered a prospective target for the prevention and treatment of vascular diseases (Luo et al. 2018). Serum amyloid A proteins (SAAs) are acute phase proteins associated with atherosclerosis. SAA-stimulation of macrophages activated NF-κB-JMJD3 signaling, which was linked to reduced H3K27me3 epigenetic markers (Yan et al. 2014). NF-κB-JMJD3 signaling also enhanced keratinocyte migration to promote wound closure. This effect was mediated by enhancing of metalloproteinases expression, motogenic growth factors, and cytokines, such as IL-12, hepatocyte growth factor, and heparin-binding epidermal growth factor (EGF) (Na et al. 2016; Odorisio 2016). NF-κB-JMJD3 signaling up-regulated the expression of matrix metalloproteinase-3 (MMP-3) and MMP-9 in injured vascular endothelial cells and promoted the blood spinal cord barrier destruction after spinal cord injury (Lee et al. 2016). The activation of NF-κB-JMJD3 signaling were increased during inflammation in microglia cells and might promote the progress of neurodegenerative diseases including amyotrophic lateral sclerosis, Parkinson’s disease and Alzheimer’s disease, and resulted in damage to surrounding neural cells (Lee et al. 2014a, b). In cytokine treated human myeloid leukemia mononuclear cells (THP-1), gene expression and proteomic analysis showed that knockout of JMJD3 inhibited the expression of key inflammatory genes regulated by NF-κB (Das et al. 2010, 2013).

#### *STAT signaling*

It has been reported that the promoter sequence upstream of the first exon of JMJD3 gene contains multiple STAT loci, and it is the reason why STAT signal transduction stimulates JMJD3 transcription (Salminen et al. 2014; Przanowski et al. 2014). JMJD3 was activated by STAT1 and STAT3 in the primary microglial rat model treated by LPS and enhanced the transcription of crucial inflammatory genes, e.g., interferon regulatory factor 7 (IRF7), CC chemokine ligand 5 (CCL5), and IL-6. STAT-dependent inflammatory genes were inhibited via the silencing of JMJD3 expression (Przanowski et al. 2014). JMJD3 was reported to link STAT signaling and Toll-like receptor (TLR) signaling in microglia: stimulation of TLR4 in microglia induced NF-κB-dependent cytokine induction, and enhancement of phosphorylation and transcriptional activity of STAT1 and STAT3 in tissues after release of these cytokines. Both NF-κB and STAT pathways up-regulated JMJD3, and JMJD3 further cooperated with them to control the production of full-spectrum pro-inflammatory mediators (Hanisch 2014). JMJD3 was reported to control the expression of genes that regulated Th1 differentiation through influencing STAT3 and STAT4 binding sites in them. JMJD3 enhanced the expression of those genes by reducing H3K27me3 level in the affected promoter. For example, JMJD3 controlled STAT3 and STAT4 to induce IL-12 gene transcription. These findings proved that JMJD3 was important in STAT3 and STAT4 induced Th1 differentiation and inflammation (Pham et al. 2013; LaMere et al. 2017).

#### *T-bet signaling*

T-bet, a Th1-specific transcription factor, is selectively expressed in Th1 cells. It is crucial in the differentiation of Th1 cells and inhibits the synthesis of Th2 cytokines by initiating Th1 genetic program (Miller and Weinmann 2010). Invariant natural killer T cells (iNKT cells) secreted diverse cytokines and influenced many types of immune responses, and protected organism from infection, inflammation and damage. T-bet was found to own the ability to recruit JMJD3 to target genes. The interaction between T-bet factor and brahma-related gene 1 (BRG1), components of Yeast mating-type switching/sucrose non-fermenting (SWI/SNF) chromatin remodeling complex, was mediated by JMJD3. SWI/SNF destroyed histone-DNA interaction and activated the expression of Th1 target genes, such as interferon gamma (IFN-γ gene via its ATPase activity. The activation of T-bet target gene induced by JMJD3 had nothing to do with its demethylase activity, suggesting that JMJD3 was a vital link between T-bet and SWI/SNF complexes (Miller and Weinmann 2009). Subsequently, it was observed that T-bet promoted H3K4me2 methylation by recruiting STAT4 and histone methyltransferases to enhance Th1 associated genes expression (Pham et al. 2013). The specific deletion of JMJD3 promoted naive T cells differentiating into Th2 helper T cells and Th17 cells, and inhibited the differentiation into Th1 helper T cells or regulatory T cells (Li et al. 2014).

#### *TGF-β*/*SMAD3 signaling*

TGF-β signaling is important in the regulation of autoimmune diseases. JMJD3 had been confirmed to play a role in systemic sclerosis, e.g., regulating T helper cell differentiation and inhibiting the expression of IFN-γ, IL-4, and IL-2 genes (David and Massagué 2018). TGF-β could signal through a number of pathways, for example, the SMAD3 pathway had a clear link to immune regulation. There is more and more evidence that JMJD3 plays a crucial role in SMAD3-mediated TGF-β signaling pathway. Progressive fibrosis was induced by the SMAD3 pathway in chronic inflammation (Rockey et al. 2015). The expression of JMJD3 was found increased in fibroblasts in systemic sclerosis (SSc) skin and in experimental fibrosis in a TGF-β/SMAD3 signaling dependent manner. Adaptor related protein complex 1 (AP1) transcription factor family has previously been proved to be crucial in the process of SSc. Fos-related antigen 2 (FRA2) is a member of the AP1 family. It was indicated that JMJD3 promoted fibroblast activation via enhancing FRA2 expression. Special silencing of JMJD3 inhibited abnormal activation of SSc fibroblasts and exert effective anti-fibrotic effects in mouse models (Bergmann et al. 2018). In osteoarthritis, TGF-β enhanced the expression of JMJD3 and increased JMJD3 targeted osteoarthritis (OA)-associated genes, and thus enhanced chondrocyte hypertrophy and matrix destruction, leading to OA progression (Yapp et al. 2016). In systemic lupus erythematosus, with characteristic of overactive T cells and over-stimulated B cells, hematopoietic progenitor kinase 1 (HPK1) expression was decreased in CD4^+^ T cells. The promoter of HPK1 which inhibited T cell-mediated immune responses had an increased level of H3K27me3 marker and reduced JMJD3 binding. Special silencing of JMJD3 increased the enrichment of H3K27me3, suggesting that expression of JMJD3 in CD4^+^ T cells contributed to the progress in pathology of systemic lupus erythematosus (Zhang et al. 2011).

#### *MAPK signalling*

ERK, JNK and p38, subfamilies of the major mitogen-activated protein kinase (MAPK) were activated by pattern recognition receptors of innate immune cells after infection or injury. MAPK enhanced the expression of multiple inflammatory genes (Arthur and Ley 2013). MAPKs are ubiquitously expressed and cover almost all aspects of the inflammatory network. Rheumatoid arthritis (RA) was regulated by JMJD3 through regulating synoviocyte-mediated fibroblast-like proliferation and joint destruction in RA. In RA, JMJD3 was activated by ERK/MAPK signaling, and the increased JMJD3 led to a lower H3K27me3 mark on the promoter of the proliferating cell nuclear antigen (PCNA) gene, which resulted in the elevated transcription of PCNA and aggressive phenotype of RA (Jia et al. 2018). In another research, JMJD3 was up-regulated by ERK/MAPK signaling, and increased JMJD3 decreased H3K27me3 levels in TLR2 promoter, which subsequently upregulated the pro-inflammatory gene of RA (Jia et al. 2018).

#### *JMJD3-IRF4 signaling*

JMJD3-IRF4 signaling also plays a role in autoimmune diseases. Studies on genetically deficient mice had shown that IRF4, CCL17 and CCR4 were essential for osteoarthritis pain, osteophyte size and optimal cartilage destruction. In the synovium, granulocyte-macrophage colony stimulating factor (GM-CSF) and IRF4 regulate CCL17 expression in macrophages. JMJD3 aggravated arthritic pain in OA through GM-CSF-JMJD3-IRF4-CCL17 pathway. Therapy targeted CCL17 and JMJD3 ameliorated both pain and osteoarthritis (Lee et al. 2018). In addition to the above diseases, JMJD3 also plays an important role in experimental autoimmune encephalomyelitis (EAE). JMJD3 deficient mice were proved resistant to EAE. *In vitro* experiments showed that JMJD3 promoted the inflammatory process of EAE by promoting the secretion of cytokines, e.g., IL-6, IFN-γ, and TNF-α by dendritic cells (DCs). By transplanting GSK-J4-treated DC into EAE mice, the clinical manifestations of EAE mice were alleviated, and the degree of inflammatory CD4^+^ T cells infiltrating the central nervous system was reduced (Doñas et al. 2016).

### **Anti-inflammatory effect of JMJD3**

#### *IL-4/STAT6/IRF4 signaling*

Macrophage polarization is closely related to the solution of inflammation, which returns to normal after infection or injury. Under environmental stresses, macrophages polarize into pro-inflammatory phenotypes, which are called M1 classical subtypes or alternately activate M2 macrophages with many anti-inflammatory properties like IRF4, STAT6 and peroxisome proliferator-activated receptor (PPAR). JMJD3 induces M2 activation. M2 macrophages secrete a variety of anti-inflammatory proteins, such as TGF-β, IL-10 and IL-4 (Murray 2017). Chemokine (C-C motif) ligand 17 (CCL17) was considered to be an M2 cytokine as IL-4 could induce it during M2 formation through JMJD3-IRF4 pathway. Importantly, this pathway had been identified in both human and mouse cells, indicating its universal relevance. In addition, inhibiting the STAT6 gene not only inhibited the production of CCL17 induced by IL-4, but also inhibited the expression of JMJD3 and IRF4 in human monocytes. In addition, STAT6, considered as a transcription factor associated with M2 differentiation, binded to the promoters of CCL17, IRF4 and JMJD3 genes (Hsu et al. 2018). Protein kinase B (Akt)/STAT6-JMJD3/IRF4 pathway was found crucial for M2 differentiation of dMϕ induced by receptor activator of nuclear factor-κB ligand (RANKL) at the maternal-fetal interface *in vitro* and *in vivo*, and the activation of Akt/STAT6-JMJD3/signaling induced decidual M2 macrophage polarization to promote smooth gestation (Meng et al. 2017). Akt/STAT6-JMJD3/IRF4 signal transduction induced polarization of M2 macrophages also promoted pulmonary fibrosis (He et al. 2016). JMJD3 expression was also enhanced in IL-4 activated bone marrow macrophages and N9 microglia, and JMJD3 induction dependented on STAT6 (Ishii et al. 2009; Satoh et al. 2010; Tang et al. 2014). Activated by Akt1/Akt2 and cAMP response element-binding protein, JMJD3 was proved to down-regulate inflammatory genes expression and microglial polarization in neuropsychiatric disorders, neurodegenerative and neuro-inflammatory (Alexaki et al. 2018).

## **JMJD3 IN INFECTIOUS DISEASES**

The innate immune response is an important determinant of resistance to infection. Accurate control of innate immune response is the key factor to maintain host immune homeostasis and ensures pathogen clearance. JMJD3 may play the opposite effect in the epigenetic regulation of different types of infections. JMJD3 enhanced the production of type I interferon in macrophage to inhibit the infection of VSV (vesicular stomatitis virus) and HSV (herpes simplex virus) (Sun et al. 2019); JMJD3 activated viral immediate early (IE) gene expression, which was crucial in anti-viral infection to inhibit human cytomegalovirus (hCMV) viral infection (Gan et al. 2017); In ameba infection, up-regulation of JMJD3 expression continued to increase bone marrow colony stimulating factor 2 receptor alpha (Csf2ra) expression and granulocyte monocyte precursors (GMPs), and prevented amoebic infection (Burgess et al. 2016); JMJD3 was found to enhance the transcription efficiency of HBV via its interaction with C/EBPα (Chen et al. 2016); JMJD3 promoted the mycobacterial infection by assisting foamy macrophages (FM)s harbor lipid bodies generation (Holla et al. 2016); JMJD3 conducived to Kaposi’s sarcoma associated herpesvirus (KSHV) invasion by noncoding polyadenylated transcript PAN RNA retained in the nucleus of host cells in infection, and decreasing the repressive H3K27me3 mark at the lytic switch protein gene promoter (Rossetto and Pari 2012). These findings indicate that JMJD3 mediate pathogen infection mainly through regulating the expression of key infection-related genes or increasing the production of key infection-related cytokines through various pathways, and provides insights into potential targeted therapeutic avenues for the treatment of infectious diseases.

### **IRF4 signaling**

JMJD3-IRF4 signaling is important for the induction of M2 macrophage polarization. Expression of JMJD3 was found increased in helminth infections. The lack of JMJD3 influenced the methylation degree of H3K27 in several genes in helminth infection. Among these, IRF4 played as a key transcription factor in M2 macrophage differentiation. Collectively, JMJD3-IRF4 was proved crucial for regulating M2 macrophage differentiation in the process of anti-helminth host reaction (Satoh et al. 2010).

### **NF-κB signaling**

TLR2-NOTCH1-phosphatidylinositol 3 kinase (PI3K)-mTOR-NF-κB-JMJD3 pathway was previously shown to play an important role in mycobacterial infection, and promoted the expression of genes associated with M2 macrophage differentiation. JMJD3 might contribute significantly to the avoidance response during mycobacterial infection, and inhibition of these pathways during infection might provide greater immunity (Holla et al. 2016). NF-κB-JMJD3 signaling was also activated by anthrax lethal toxin treatment in a macrophage cell line, RAW 264.7 cells, which might be necessary for the activation of genes to change into intoxication-resistant cells (Das et al. 2010).

### **Wnt signaling**

Wnt signaling pathway is a crucial network in viral mediated cell signaling. The main mechanisms of the interaction between virus and Wnt pathway appear to be epigenetic modification of Wnt gene (Doorbar et al. 2015). Human immunodeficiency virus type-1 (HIV-1) is a lentivirus. Wnt signaling were reported down-regulated in HIV patients in a recent article on transcriptome analysis of HIV-positive and hepatitis C virus (HCV)-positive patients (Wu et al. 2011). The global gene expressions of intestinal epithelial cells in the same animal before and after swine influenza virus (SIV) infection 21 days and 90 days was analyzed, and the genes expression levels of Wnt signaling components were significantly decreased. Increased expression of JMJD3 at 90 DPI indicated that epigenetic mechanisms involved in histone modifications might play a role in inhibiting of the Wnt pathway (Mohan et al. 2013).

## **JMJD3 IN CANCER**

Based on existing researches, the role of JMJD3 in cancer is quite complex and highly controversial. JMJD3 was found to play a suppressive role in cancers of colorectum, lung, liver, hemopoietic system, e.g., non-small cell lung cancer (NSCLC), glioma, B-cell lymphoma and pancreatic ductal adenocarcinoma. However, JMJD3 was also reported to play a carcinogenic role in other types of cancer in kidney, breast, prostate, skin, hemopoietic system, melanoma, renal cell carcinoma, Hodgkin’s lymphoma (HL), myelodysplastic syndrome (MDS), esophageal squamous cell carcinoma, ovarian cancer. Here we discuss the regulation mechanisms of JMJD3 in cancer and potential therapeutic avenues targeting JMJD3. Many studies have revealed surprisingly roles of JMJD3. The unique physiological roles of JMJD3 in leukemia is built on the demethylase-dependent and demethylase-independent manners of JMJD3 on histone methylation, its post translational modifications, and its participation in different complexes as well as *in vivo* modeling of the mutations.

### **The role of JMJD3 in promoting EMT**

#### *TGF-β signaling*

TGF-β is a unique multipotent cytokine, which inhibit cell proliferation and induce apoptosis in the early stage of tumor growth and induce epithelial-mesenchymal transition (EMT) in the late stage of tumor growth (Wendt et al. 2012). In typical TGF-β signal transduction, the binding of TGF-β ligands to type II TGF-β receptor enabled the polypeptide to transphosphorylate and activate type I TGF-β receptor, while type I TGF-β receptor binded to phosphorylate and activate receptor-related Smad family transcription factors Smad2 and Smad3 (David and Massagué 2018). The antagonistic effect of Smad2/3 signal transduction of JMJD3 protein (TGF-β related extracellular ligand) on the inhibition of multiple comb related genes in embryonic stem cells suggested that Smad proteins and chromatin dynamics at gene promoters cooperated with each other to release gene inhibition (Kim et al. 2011). It was demonstrated that JMJD3 also stimulated SNAI1 expression and played a crucial role in TGF-β-induced EMT in mammary epithelial cells. JMJD3 was proved to be induced by TGF-β and silencing of JMJD3 inhibited the EMT induced by TGF-β. On the contrary, the overexpression of JMJD3 enhanced the expression of mesenchymal genes like SNAI1 and promoted EMT. Further study revealed that JMJD3 inhibited the EMT induced by TGF-β mainly via promoting SNAI1 expression in invasive breast carcinoma. The knockdown of JMJD3 significantly inhibited the EMT and invasion of breast cancer cells (Ramadoss et al. 2012). Overexpression of JMJD3 was also proved to be associated with Poor prognosis in ovarian cancer patients. Overexpression of JMJD3 promoted EMT, proliferation, invasion and migration of epithelial ovarian cancer cells *in vitro*, and enhanced metastatic capacities *in vivo* by promoting the growth factor-β (TGF-β1) expression (Liang et al. 2019).

### **Carcinogenic role of JMJD3**

#### *BMP signaling*

Disorders in signal transduction of bone morphogenetic protein (BMP) signaling is confirmed to be carcinogenic in humans and mice. BMP signaling promotes cancer by activating the NF-κB signaling induced by several stimuli. JMJD3 was found to interact with SMAD1 and SMAD4, crucial transcription factors in BMP signaling and upregulated CCL2 and tissue factor pathway inhibitor 2 (TFPI2) in melanoma cells. JMJD3 also confered self-renewal ability in melanoma cells by up-regulating expression of stanniocalcin-1 (STC1) via NF-κB pathway. JMJD3 enhanced the progression and metastasis of melanoma by activating NF-κB and BMP transcriptional networks (Park et al. 2016).

#### *MAPK signaling*

MAPKs are important signaling components in the transformation of extracellular stimuli into a wide range of cellular responses. Expression of ERK1 and ERK2 which were activated by mitogens were found enhanced in human tumors. This finding had promoted the development of inhibitors targeted this pathway in cancer treatment (Wagner and Nebreda 2009). JMJD3 was found recruited to gene loci which encoded components of MAPKs, including FOS and ELK1, and enhanced the expression of these genes in a demethylase-dependent manner to mediate multiple myeloma cell growth and survival (Ohguchi et al. 2017). Overexpression of JMJD3 was proved independently associated with poor prognosis in esophageal squamous cell carcinoma patients. Suppression of JMJD3 expression inhibited esophageal squamous cell carcinoma cells’ growth rate, and JMJD3 overexpression promoted esophageal squamous cell carcinoma cell proliferation. This phenomenon was associated with Ras/MEK pathway (Li et al. 2019).

#### *NOTCH1 signaling*

NOTCH1 acts as a transcription factor that directly transduces signals from extracellular environment into nucleus and changes transcriptional pattern of specific target genes. Activation of NOTCH1 in the thymus was found essential for specification of early T cell fate and thymocyte development in the hematopoietic system (Belver and Ferrando 2016). JMJD3 was proved to be a component part of the NOTCH1 transcriptional complex, as it interacted directly with NOTCH1 and mastermind-like protein 1 (MAML1). The NOTCH1-JMJD3-MLL complex was proved to enhance the cell growth-promoting effects of the NOTCH1 transcriptional program in acute lymphoblastic leukaemia of mice. Ablation of JMJD3 gene in T cell acute lymphoblastic leukemia (T-ALL) resulted in the decrease of leukemia cells in peripheral blood, the decrease of leukemia cell infiltration in spleen and liver, and the increase of survival rate in the recipients. Targeting JMJD3 activity was proved therapeutic potential in T-ALL (Ntziachristos et al. 2014). Jin et al. reached a unanimous conclusion in the promoting effect of JMJD3 in T-ALL, and proved that the ubiquitin-specific protease 7 (USP7) interacted with NOTCH1 and JMJD3 to stabilize their expression ability. Chemical inhibition of USP7 alone or in combination with JMJD3, using the GSK-J4 compound, blocked leukemia growth *in vivo* (Jin et al. 2019).

JMJD3 also plays a carcinostatic or carcinogenic role through activating key target genes by demethylation. In neuroblastoma, the failure of neural crest cell precursored to differentiate and the activation of key oncogene was the root cause of neuroblastoma. Inhibiting the histone JMJD3 with GSK-J4 had profound regulating efficiency on crucial differentiation genes and pathways, including reducing v-myc myelocytomatosis viral related oncogene (MyCN) expression. Therefore, JMJD3 had a promising therapeutic potential in treating high-risk neuroblastoma (Lochmann et al. 2018). JMJD3, located on chromosome 17, was very close to p53, a tumor suppressor. Mutations of these two genes were common in human cancers. JMJD3 was also reported to have functional interactions with p53 (Ene et al. 2012; Williams et al. 2014). JMJD3 also activated key oncogenes in gene of phosphate and tension homology deleted on chromosome ten (PTEN) and androgen receptor (AR) pathways participated in prostate cancer. JMJD3 was a potential new biomarker for prognosis (Daures et al. 2018). The B cell receptor (BCR) signaling pathway represents a key component of normal B cell survival during the whole development. Dysregulated BCR signaling has been proved to be crucial in lymphogenesis and tumor survival in many B-cell non-Hodgkin’s lymphoma (NHL). JMJD3 was found to promote phosphorylation of proteins mediating BCR signaling and up-regulate proto oncogene B-cell lymphoma/leukemia 6 (Bcl6) levels to facilitate diffuse large B-cell lymphoma (DLBCL) progression. This effect was reversed by the specific inhibitor of JMJD3, GSK-J4 (Valla et al. 2018). In promyelocytic leukemia/retinoic acid receptor α (PML-RARα)-positive leukemic cells, JMJD3 was found activated by the PML-RARα fusion protein and enhance expression of homeobox (HOX) gene. Abnormal expression of Hox gene was considered to be associated with leukemia, and affected disease progression and survival in leukemia patients. The combination of GSK-J4 with all-trans retinoic acid (ATRA) significantly increased the apoptosis of PML-RARα positive cells (Rejlova et al. 2018). Inhibiting JMJD3 in diffuse intrapontine glioma (DIPG) orthotopic grafts was also proved to reduce the growth of tumors and significantly prolonged the survival time of animals. GSK-J4 treated tumors showed decreased proliferation and increased apoptosis, compared to untreated control tumors (Lulla et al. 2016). JMJD3 was also activated by EBV oncogene and latent membrane protein 1 (LMP1) in GC B cells, which were potential ancestors of HL. In germinal B (GC-B) cells, JMJD3 was also proved to be associated with the differentially expressed genes in HL. The deletion of JMJD3 enhanced the trimethylation of H3K27 on these genes (Anderton et al. 2011). JMJD3 was also found to promote expression of key genes involved in DNA replication and cell cycle phase transition, such as minichromosome maintenance deficient 4 **(**MCM4), heat shock protein 90-alpha 1 (HSP90AA1), flap endonuclease-1 (FEN1), MCM3, PCNA, and oncogene HOX genes to promote the progression of acute myeloid leukemia (AML) in mouse. GSK-J4 combined with first-line chemotherapy drug Ara-C has synergistic inhibitory effect on colony formation of AML cells in mice (Li et al. 2018). The increase of JMJD3 was also reported to be unfavourable prognosis in human clear cell renal cell carcinoma (ccRCC), which might be associated with the up-regulation of oncogenes (Wang et al. 2017). In myeloid malignancies, JMJD3 impaired osteoclast formation through increasing the level of H3K27me3 on the nuclear factor for activated T cells 1 (NFATc1) promoter to promote myeloid malignancies (Rohatgi et al. 2018).

### **Tumor suppressive effect of JMJD3**

#### *Wnt signaling*

WNTs is the broad pathological basis of humans and is critical for cancer progression, including tumor growth, tumor initiation, cellular senescence, cell differentiation, cell death, and the metastasis (Clevers and Nusse 2012). Components of Wnt/β-catenin pathway are often overexpressed or under-expressed in different kind of human cancers, which is often linked to epigenetic inhibition or activation of gene promoters. Wnt/β-catenin pathway was found carcinogenic in colon and breast cancer (Nusse and Clevers 2017). Accumulation of catenin in nuclear was often viewed as increased Wnt/β-catenin signaling activity, which was proved to be associated with poor prognosis of colon cancer. It was proved that 1,25(OH)_2_D_3_ induced JMJD3 in colon cancer cells and antagonise the Wnt/β-catenin signaling by promoting a direct VDR-β-catenin interaction and nuclear export of β-catenin, which together led to inhibition of the transcriptional activity of β-catenin/T-cell factor (TCF) complexes. JMJD3 gene knockout blocked the antagonision of 1,25(OH)_2_D_3_ to the Wnt/β-catenin signaling, and it was attributed to the negative influence of JMJD3 on VDR-β-catenin interaction and nuclear export of β-catenin (Pereira et al. 2011, 2012).

#### *INK4a-ARF signaling*

P16-INK4a and ARF are encoded by INK4a/ARF locus, and respectively regulate the RB and p53 pathways. Recent studies had determined that both proteins suppressed tumor activity significantly *in vivo* (Sharpless 2005). JMJD3 binded to the inhibitor of cyclin dependent kinase 4a (INK4a)/ARF locus, and induced cell cycle arrest depended on P16-INK4a in mouse embryonic fibroblasts (Barradas et al. 2009). The INK4a/ARF locus was found to up-regulate JMJD3 to promote the neurofibroma oncogene induced senescence, and JMJD3 further blocked Schwann cell proliferation through the p19 ARF/p53 and p16 INK4a/Rb pathways (Gomez-Sanchez et al. 2013). JMJD3 was also found to promote THP-1 cell differentiation into monocytes and inhibited progression of leukemia (Park et al. 2018). JMJD3 functioned as a tumor suppressor gene in renal cell carcinoma (RCC), while in bladder cancer its over expression might promote cancer formation and progression (Hong et al. 2017).

## **JMJD3 IN DEVELOPMENTAL DISEASES**

JMJD3 is directly involved in the embryogenesis of the three embryonic layers of developing vertebrate: developing vertebrate endoderm, mesoderm and ectoderm. The demethylation of H3K27 is removed in a tissue and cell-specific manner during differentiation. JMJD3 level was higher in the adult retina, in vivo inhibition of histone demethylase blocking by GSK-J1 influenced cell proliferation, maturation, apoptosis induction, and specific cell determination. In the abnormal development and diseases of the central nervous system, JMJD3 was proved to be a biological target in therapeutic strategies nervous system (Raeisossadati et al. 2019); Inhibition of JMJD3 increased the H3K27me3 content of podocytes and attenuated glomerular disease in adriamycin nephrotoxicity, and diabetes (Majumder et al. 2018). JMJD3 played a regulatory role in Hox expression during axial patterning of mice (Naruse et al. 2017). During murine tooth germ development, JMJD3 was expressed in a spatial-temporal manner, and regulated the cell differentiation in tooth organ development (Zheng et al. 2014). JMJD3 was an important activator of neurogenesis from adult subventricular zone (SVZ) neural stem cells (NSCs). The expression level of JMJD3 was upregulated in neuroblasts, and JMJD3 deletion targeted and JMJD3 deletion impaired neuronal differentiation in both developing and adult mice impaired (Park et al. 2014); JMJD3 in the bronchopulmonary dysplasia (BPD) rat model, JMJD3 was found significantly reversed the hyperoxia-induced down-regulation of RUNX3, which was associated with pulmonary epithelial and vascular development in AT2 cells (Zhu et al. 2015). *In vivo* pharmacological blocking of JMJD3 influenced maturation, cell proliferation, apoptosis induction, and specific cell determination. The role of JMJD3 in the abnormal development and diseases can be explored in the therapeutic strategies.

### TGF-β/SMAD3 signaling

TGF-β/SMAD3 signaling is one of the earliest signals to work. TGF-β/SMAD3 signaling is important in the embryonic development of vertebrates, and is essential for many developmental processes. JMJD3 was found recruited to target genes by the Nodal-Smads2/3 (Smad2 and Smad3) signalling, thereby counteracting the inhibition via polycomb. SMADS2/3 binded to JMJD3 and JMJD3 was recruited to chromatin in a manner depending on active Nodal signaling. The early stage of embryonic differentiation was driven by hard-wired pathways inducing specific fates. Nodal, a member of the TGF-β superfamily, utilized transcription factors FOXH1, SMAD2/3, and SMAD4 to drive the expression of target genes, thereby controlled the differentiation of endoderm. JMJD3 was also recruited into these promoters, suggesting a genome-wide protection mechanism in multiple promoters. The correlation between SMAD2/3 binding, univalent formation and transcriptional activation suggested that SMAD protein coordinated with chromatin at key promoters to drive endodermal regulation (Kim et al. 2011). The TGF-β development program also requires JMJD3 to proceed. Whole genome analysis showed that JMJD3 binded to the promoter regions of SMAD3 factor to control a panel of TGF-β-responsive the expression of genes during neurogenesis. Then, JMJD3 interacted with Ser-2 phosphorylation Pol-II in the extension complex, which significantly enhanced the transcriptional process, as JMJD3 demethylated H3K27me3 on the genome (Estarás et al. 2012; Estarás et al. 2013).

### T-bet signalling

T-box signaling is confirmed important in development. Deletion of eomesodermin (Eomes or Tbr2), the T-box transcription factor, in ectodermal cells resulted in embryos that entirely lack of definitive endoderm (Arnold et al. 2008). Tbx3 and Eomes, which were embryonic T-box transcription factors, were proved crucial for endoderm formation. Eomes usually maintained a transcriptional balance in embryonic stem (ES) cells. During early stage of endodermal differentiation, Tbx3 would bind to JMJD3 in the enhancer region of Eomes to promote its expression. The DNA looping of Eomes allowed the activation loop to maintain the fate of the endoderm and preventing abnormal development (Kartikasari et al. 2013).

### Wnt signaling

The Wnt signaling is the primary regulator of the development of the entire animal kingdom. Wnt signaling has become a basic growth control approach in early animal evolution. The mutated components of Wnt pathway are responsible for a variety of growth-related pathologies. It has been proved that deletion of JMJD3 in embryonic stem cells does not affect its self-renewal, but significantly impairs mesoderm formation during differentiation. JMJD3 was recruited to the promoter of the Brachury gene, which was crucial to the differentiation of mesoderm, and removed the inhibitory H3K27me3 marker and additionally promoted β-catenin-dependent gene activation following Wnt stimulation. In conclusion, the β-catenin depended Wnt signaling pathway played a role in the differentiation of embryonic stem cells through JMJD3 (Ohtani et al. 2013). JMJD3 also epigenetically activated Wnt5a activation by decreasing H3K27me3 marks in odontogenic differentiation (Zhou et al. 2018). Wnt family members and their inhibitors become transduced into changes in the 3-dimensional structure of chromatin that locally influences expression of cardiogenic genes. In the process of cardiac fate, JMJD3 demethylation of H3K27 residues was found needed for Wnt-stimulated nuclear β-catenin to activate certain mesoderm genes (Willems and Mercola 2013).

### RAS/MAPK signaling

The RAS/MAPK signalling controls proliferation, differentiation and survival of cells and is an important signaling cascade. The RAS genes encode a 21 kDa protein family and compose an important hub for many pathways of survival, differentiation, proliferation, and senescence. Signaling of RAS-GTPases-RAF-MEK-ERK, the first identified MAPK cascade, is crucial for development (Inoue et al. 2017). JMJD3 has been proved to be involved in MAPK signaling pathway. Its specific inhibitor GSK-J4 was proved partly rescued the esophageal dilation, gastrointestinal abnormalities, growth retardation and in BRAF Q241R mutation mice in which the BRAF mutations resulted in the activation of the RAS/MAPK pathway. JMJD3 was suggested to promote the pathological process of RAS opathies (Cao et al. 2017).

### NF-κB signaling

The NF-κB family is known as a key transcription family primarily for its contribution to the immune responses and infected vertebrates. The NF-κB signalling pathway leads to the accumulation of NF-κB to the nucleus to function as a transcription factor. Change of NF-κB members in various animal models resulted in severe developmental defects even embryonic lethality (Espín-Palazón and Traver 2016). NF-κB is also proved to be a crucial role in hematopoiesis. It was reported that both classical and non-canonical NF-κB activation regulated hematopoietic stem cells (HSC) homeostasis and intrinsic function (Stein and Baldwin 2013). Expression of JMJD3 was found up-regulated in MDS CD34^+^ cells in human myelodysplastic syndrome. Natural immune stimuli derived from chronic inflammation, hematopoietic cells, or stroma activated NF-κB in MDS CD34^+^ cells in a paratactic or autocrine manner. NF-κB activated many other effectors, such as JMJD3. A feedback loop was formed between NF-κB, JMJD3 and the innate immune effectors. Signal transduction through this feedback loop collaborated with other lesions in MDS cells and played a role to bone marrow failure and conversion to AML characterized by MDS. JMJD3 might influence the determination of hematopoietic lineage and contribute to the progression of MDS (Wei et al. 2013). DNA and histone methylation might interact to promote Huntington’s neuron differentiation and enhance neuronal progenitor-associated genes, which were essential for neuronal fate acquisition (Park et al. 2014). The expression of JMJD3 was found lower in human induced pluripotent stem cells derived from a HD patient (HD-hiPSCs) than H9 human embryonic stem cells (H9-hESCs). This led to the hypothesis that an effect on cell identity formation might be a seed of developmental epigenetic defects associated with Hodgkin’s disease (HD) (Baronchelli et al. 2017). JMJD3 was also found to up-regulate the expression of RUNX3, which was associated with pulmonary development in alveolar type 2 (AT2) cells treated with hyperoxia. It was suggested that JMJD3 may alleviate bronchopulmonary dysplasia (BPD) by activate genes related to development (Zhu et al. 2015). JMJD3 decreased H3K27me3 level to promote porcine nuclear reprogramming, and enhanced both porcine somatic cell nuclear transfer (SCNT) and induced pluripotent stem cells (iPS) efficiency. JMJD3 reduced developmental disorders (Pinheiro et al. 2015). In axial skeletal formation of mice, demethylase activity of JMJD3 played an important role in axial patterning by decreasing Hox gene expression, which led ultimately to morphologic malformations (Naruse et al. 2017).

## **JMJD3 IN AGING RELATED DISEASES**

Aging is caused by a variety of mechanisms, including oncogenic signals, telomere shortening, and DNA damage. Induction of aging by oncogenes is one of the important mechanisms of aging (Bianchi-Smiraglia et al. 2017). Epigenetic silencing of gene loci encoding aging pathway effectors usually occurs in normal dividing cells. Numerous studies have confirmed that JMJD3 plays a key role in activating important genes during aging. JMJD3 plays a critical role in the aging process via TGF-β, INK4-box and p53 signaling. JMJD3 regulated the expression of factors that manipulated p16, senescence-related secretory phenotypes (SASP), p53 and TGF-β/SMADs which acted as transcription inhibitors and/or activators, allowing the expression of genes that promote cell cycle arrest (Tamgue and Lei 2017). More and more literatures show that the p53 signaling network is related to cell senescence and aging process. JMJD3 represents the primary risk factor in major aging related diseases such as cancer, diabetes, cardiovascular disorders, and neurodegenerative diseases. Especially, JMJD3 prevents cancer formation via oncogene-induced senescence (OIS), since RAS and p53 signaling stimulate JMJD3 function (Salminen et al. 2014). JMJD3 mediated cell senescence might play an important role in the anticancer activity of calcitriol in kidney cancer through the up-regulation of cell senescence marker p16INK4A (Shen et al. 2017); ectopic expression of JMJD3 promoted cellular senescence and senescence-associated heterochromatin foci (SAHF) formation in WI38 cells, which helped to stabilize the senescence state induced by anticancer drugs (Zhao et al. 2015); Overexpressing wild-type JMJD3 (JMJD3wt) activated senescence-associated secretory phenotype (SASP)-associated genes in glioma cell lines, and enhanced glioma cells invasiveness associated with increased expression of proteases typical of the SASP (Perrigue et al. 2015); in neurofibroma, the induction of JMJD3 epigenetically activated the Ink4a/Arf-locus, forcing Schwann cells towards replicative senescence, and prevented malignant transformation of neurofibromas (Gomez-Sanchez et al. 2013). Inhibition or overexpression of JMJD3 may be a potential therapeutic tool for aging-related diseases.

### p53 signaling

The P53 transcription factor is an antioncogene that controls apoptosis, cell cycle DNA repair, autophagy, and many stress responses to maintain intracellular homeostasis. Recent studies had shown that methylation status of lysine in p53 protein plays a key role in displaying its function (West and Gozani 2011). JMJD3 was reported to interact with p53 protein and promote p53 accumulation in nucleus in process of differentiation in mouse neural stem cells. The nuclear accumulation of p53 was influenced by alternative reading frame (ARF) expression which was demethylation-dependent and induced by JMJD3. ARF protein was coded by INK4-ARF box, which enhanced the degradation of the p53 inhibitor, murine double minute (MDM2) protein, and thus promoted the stability of p53 (Solá et al. 2011). JMJD3 was also observed to induce the nuclear translocation of p53 in glioblastoma stem cells (GSC) and HEK293 cells, and then p53 promoted the expression of p21 gene. The p21 protein was a cyclin-dependent kinase inhibitor, which induced cell aging and the expression of many genes related to aging-related diseases (Ene et al. 2012). p53 had been confirmed to be less effective during aging, which might be deleterious and led to age-related effects that affected genomic integrity, autophagy, and mitochondrial energy metabolism. In p53-deficient mice, accelerated immune aging was confirmed to be associated with phenotypes encountered (Rufini et al. 2013; Wei and Ji 2018).

### INK4 box signaling

The INK4 box, also named INK4b-ARF-INK4a, encodes p15INK4b, ARF and p16INK4a, which are three oncogene products. INK4a and INK4b are cyclin D dependent kinase inhibitor and ARF protein is an activator of p53-dependent cell-cycle arrest. All of them inhibited cell proliferation and was found increased in senescent cells, and the inhibition or mutation of INK4 locus was related to multiple cancers (Popov and Gil 2010). The control of INK4 box activity was confirmed in a strict epigenetic regulation (Simboeck et al. 2011). JMJD3 played an important role in demethylating the H3K27me3 in the promoter of INK4a and ARF gene and activating their expression during cellular stress. JMJD3 was recruited to INK4a locus and led to the expression of p16, which in turn led to irreversible growth arrest during cell senescence in normal fibroblasts (Agger et al. 2009; Barradas et al. 2009). The process how JMJD3 was recruited into the INK4 box was not clear. One possibility was that JMJD3 interacted with H3K4 methyltransferase, MLL1, and then activated epigenetic marker in the INK4 box (Agherbi et al. 2009). These studies indicated that INK4 box genes could be activated by JMJD3 via replacing the polycomb complexes with H3K4 methyltransferase under cellular stress, and led to cellular senescence. P16 is a tumor suppressor gene encoded by the INK4a gene locus. This locus was also confirmed to be regulated by the methylation status of H3K27 (lysine 27 of histone H3), and its gene expression status was controlled by JMJD3 (Bracken et al. 2007). JMJD3 upregulated p16 to inhibit the reprogramming of MEFs to become iPS cells (Zhao et al. 2013).

JMJD3 also exerted the function of restraining senescence under the stimulation of certain specific factors. Hypoxia significantly increased the expression of JMJD3, and then JMJD3 was recruited to the eNOS promoter, and reversed the inhibition of endothelial genes. JMJD3 was also proved to be essential for proangiogenic cells survival and inhibition of JMJD3 induced cell death and senescence (Williams et al. 2014). JMJD3 was also found to up-regulate mammalian mitochondrial unfolded protein response (UPRmt) core components and downstream mitochondrial chaperones to promote mammalian longevity (Merkwirth et al. 2016). Isoproterenol (ISO) stimulated JMJD3 expression and JMJD3 overexpression promoted cardiomyocyte hypertrophy by promoting the expression of hypertrophy marker genes like β-MHC. JMJD3 deficiency or its inhibitor GSK-J4 suppressed ISO-induced cardiac hypertrophy (Guo et al. 2018).

## **CONCLUSIONS AND PERSPECTIVES**

**Figure 3:**
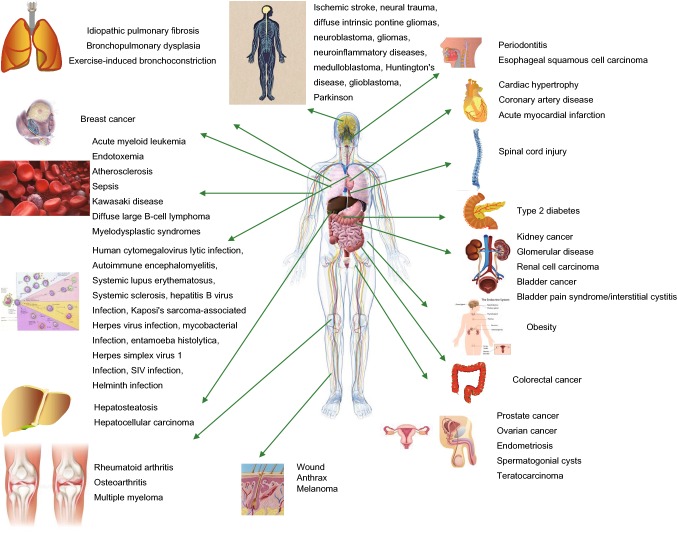
**Schematic diagram illustrating human diseases associated with JMJD3**. This map highlights those biological processes that have been proven to be related to human pathology (see text for references), and indicates that JMJD3 has been proven to be a therapeutic target or a biomarker of disease or disease progression

JMJD3 is a specific demethylase of H3K27me3 which acts on and represents as an inhibitory epigenetic histone marker. The demethylase activity of JMJD3 remains to be fully studied, and its interaction with other chromatin-modified complexes is the key to its function *in vivo.* As JMJD3 stimulates a wide range of genes involved in inflammatory agents, development, cancer, senescence and so on (Fig. 3). Activation of JMJD3 is an important host response against environmental and cellular stress. However, excessive expression of JMJD3 leads to uncontrolled transcription and destructs the nuclear structure by decreasing the level of H3K27me3, which is a key epigenetic marker in maintaining the genome’s 3D organization. It is important to emphasize that JMJD3 plays as a double-edged sword according to the cellular environment, for example, pro-inflammatory and anti-inflammatory response. Correspondingly, JMJD3 plays a dual role in many other physiological or disease processes.

JMJD3 is increasingly linked to human diseases, and it is feasible and effective to develop therapies for multiple diseases based on JMJD3. GSK-J1 has been identified as a specific inhibitor of JMJD3 catalytic site. This small molecule inhibitor was further modified by adding cell penetrating ethyl esters to produce GSK-J4, which inhibited LPS-induced cytokine expression in macrophages of healthy volunteers (Kruidenier et al. 2012). GSK-J4 was further shown to decrease airway smooth muscle cells (ASMCs) proliferation and migration in the contractive model of human ASMCs (Yu et al. 2018). GSK-J4 was also found efficient in blocking the growth of neuroblastoma cell and GSK-J4 and RA combination inhibited patient-derived xenograft models of high-risk neuroblastoma growth *in vivo* more (Lochmann et al. 2018). Treatment with GSK-J4 decreased membrane proteinase 3 (mPR3) expression which played a critical role in pro-inflammatory cytokine production in peripheral blood neutrophils of septic patients (Chen et al. 2018). GSK-J4 was also proved to reduce the proliferation and colony forming ability of human primary AML cells and cell lines. In another study, GSK-J4 treatment significantly induced apoptosis and cell cycle arrest of Kasumi-1 cells, and the expression of the carcinogenic HOX gene was eliminated by GSK-J4. The disease progression in human AML xenotransplantation mice was attenuated by treatment of GSK-J4 and demonstrated synergistic effects with cytosine arabinoside (Cribbs et al. 2018). In human breast cancer cells, GSK-J4 markedly inhibited the expression of stemness-related markers and the self-renewal capacity, expansion, proliferation of them. GSK-J4 was considered to be a prospective agent targeting breast cancer stem cells (BCSCs) (Yan et al. 2017). GSK-J4 had therapeutic applications for neuroinflammatory diseases for it suppressed important inflammatory genes expression in primary microglial (Das et al. 2017). GSK-J4 reduced type 1 diabetes incidence by protecting insulin-producing cells and human islets from cytokine-induced apoptosis and increasing expression of insulin gene and glucose-stimulated insulin secretion (Backe et al. 2018). GSK-J4 also induced AKT activation and cell cycle arrest in human THP-1 cells treated with LeTx (Ha et al. 2017). Treatment with GSK-J4 in human large B-cell lymphoma cell lines predominantly resulted in down rage of B-cell receptor signaling and BCL6 and introduced apoptosis of large B-cell lymphoma cells (Mathur et al. 2017). The role of JMJD3 in cancer is quite complex and remains unclear, and its function may be at the intersection of many pathways facilitated by dysfunction (Li et al. 2018). In the treatment of GSK-J4 on osteoporosis, the JMJD3 specific inhibitor was found to own anti-inflammatory effect on NK cell subsets of peripheral blood or tissue from RA patients and inhibited bone resorption on osteoclast formation, indicating that JMJD3 inhibitors had wide clinical application value (Cribbs et al. 2018). GSK-J4 was also found to inhibit the expression of OA-related genes PTGS2, MMP13 and alleviate cartilage damage in human bone marrow mesenchymal stem cells, providing a new way for the treatment of OA (Yapp et al. 2016). In addition to the use of small molecule inhibitors, a new technology (regular interval short palindrome repeat aggregation)/gene editing based on Cas9 (Hsu et al. 2014) can be used to delete JMJD3 for therapeutic applications. This kind of gene editing can use RNA-guided DNA recognition to precisely edit genes of interest with minimal non-target effect. This technique can also be used as a screening method to identify downstream effectors of JMJD3 and identify which genes are necessary for mediating JMJD3 responses. Complete ablation of JMJD3 may have a far-reaching effect on cells and tissues than small molecule inhibitors in human diseases. However, as JMJD3 involves in a variety of normal physiological processes, complete inhibition of JMJD3 has non-targeting and side effects. Therefore, designing cell-specific agonists or antagonists will help control any non-specific effects. JMJD3 targeted therapy relies highly on tissue and disease types. As JMJD3 controls the disease process by regulating the expression of key genes in the diseases, JMJD3-targeted therapy may own high clinical application prospects in gene mutation and gene defect diseases. While we are only at the dawn of our studies of H3K27Me3 homeostasis, much progress has been made in our understanding of JMJD3 functions since JMJD3 is identified, in 2007, as H3K27Me3 demethylases. The discovery of other key molecules relying on JMJD3 will further deepen our understanding on the role of JMJD3 in human diseases. In addition, further studies are needed to find out whether JMJD3 has other intracellular localization besides nucleus and to determine the alternative function of JMJD3 in cells. The degradation mechanism of JMJD3 RNA and protein has not been determined yet, but it may be helpful to regulate JMJD3 level and downstream histone demethylase dependent and non-dependent pathways. At present, the signal pathways and the roles of JMJD3 in them are still poorly understood. GSK-J1 and similar drugs can only be used in preclinical mouse models of leukemia in combination with classic chemotherapy and radiation therapy to evaluate the effects of demethylase inhibition in cancer. The clinical application of JMJD3 in the treatment of human diseases is still in its infancy and its more potential remains open ended.
